# Small bowel hemangioma diagnosed with laparoscopy: Report of two pediatric cases

**DOI:** 10.4103/0972-9941.30684

**Published:** 2007

**Authors:** A E Jones, B H Ainsworth, A Desai, T T Tsang

**Affiliations:** Department of Paediatric Surgery, Norfolk and Norwich University Hospital NHS Trust, Colney Lane, Norwich, NR4 7UY, UK

**Keywords:** Hemangioma, laparoscopy, pediatric, small bowel

## Abstract

Hemangiomas of the small bowel are rare tumors that often present with gastrointestinal bleeding. Diagnosis can be difficult and exploratory laparotomy has often proved to be the only method with which to determine the presence and location of these tumors. We report two cases of small bowel hemangioma in children aged 10 and 7 years, in which the diagnosis was made by laparoscopy. Laparoscopy identifies the affected segment of bowel and allows delivery to a minimally extended umbilical port site. The avoidance of an open laparotomy helps to reduce post-operative analgesic requirement and achieves an early return of bowel function.

## INTRODUCTION

Hemangiomas of the gastrointestinal tract are rare and account for only 0.05% of all intestinal neoplasms.[1] They are commonly found within the small bowel and comprise 7-10% of all benign tumors.[1] The commonest location within the small bowel is the mid-jejunum.[1] They have a tendency toward multiplicity with solitary tumors being extremely rare.[1,2] Hemangiomas have an association with systemic angiomatoses such as blue rubber bleb naevus syndrome, Maffucci's syndrome, and Klippel-Trenaunay-Weber syndrome. Grossly, an intestinal hemangioma is usually soft and polypoid; it is red, blue or wine-colored, and varies in size from a few millimeters to a large polypoid mass projecting into the lumen or infiltrating the bowel wall.[1]

Hemangiomas of the small bowel usually present with intestinal bleeding. This may be acute with massive blood loss which can be life threatening or chronic with anemia as in our two cases. Other forms of presentation include intussusception,[2] small bowel obstruction and perforation.[1,3] Diagnosis of small bowel hemangiomas can be difficult and exploratory laparotomy has often proved to be the only method with which to determine the presence and location these tumors.[1,3] We report two cases of small bowel hemangioma in children aged 10 and 7 years in which the diagnosis was made by laparoscopy.

## CASE REPORT

**Figure 1:**
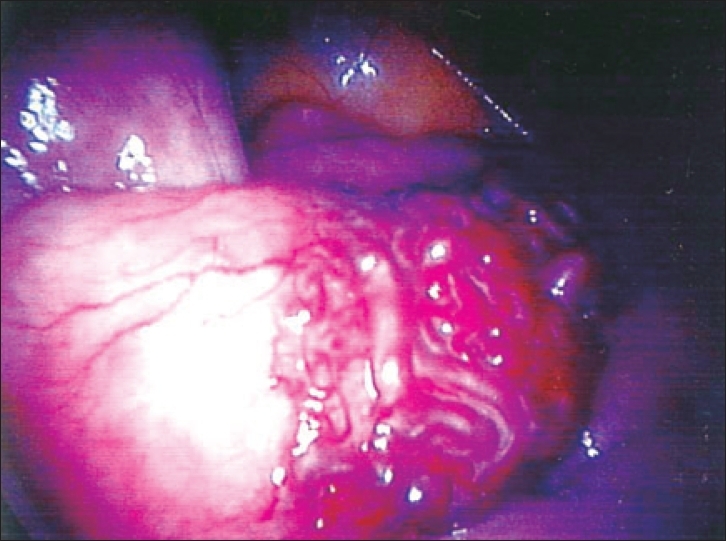
Laparoscopic view of the jejunal hemangioma

**Figure 2:**
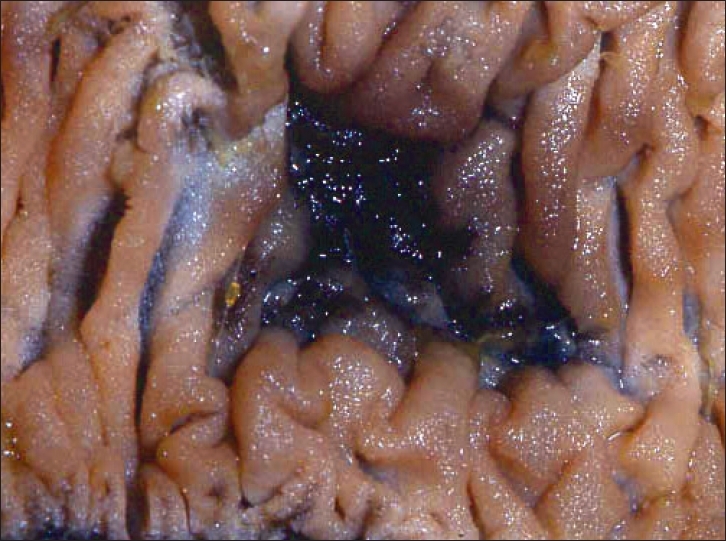
Opened specimen showing ulcerated haemangioma on the mucosal side of the jejunum

The first patient was a 10-year-old girl who was under investigation for recurrent abdominal pain and iron deficiency anemia requiring blood transfusions. Physical examination was unremarkable. Blood count revealed hemoglobin of 6.8 g/dL, mean cell volume (MCV) of 72. Double contrast barium follows through and Technetium 99 meter pertechnetate (Meckel's) scan were normal. In addition, anti-endomysial and autoimmune antibody screens were normal. However, fecal occult blood tests were positive on a number of occasions. Upper and lower gastrointestinal endoscopies were arranged, however, before these investigations could be performed, the patient presented acutely with collapse and melaena. Following fluid resuscitation, she underwent urgent oesophago-gastro-duodenoscopy (OGD) and colonoscopy, both of which revealed no abnormality. Laparoscopy was undertaken at the same time and revealed a 2 cm vascular malformation of the jejunum [Figures 1 and 2], which was resected with primary jejenal end-to-end anastomosis via mini laparotomy performed by extending the umbilical trocar incision. She made an uneventful recovery and follow up at 6 months revealed a normal blood count and hematinics. Histology of the resected bowel confirmed a jejunal vascular malformation.

The second patient was a 7-year-old girl who initially presented with a three weeks history of lethargy, anorexia, and abdominal pain. Physical examination was normal. On admission, her hemoglobin was 4 g/dL with an MCV of 62 and required blood transfusion. Fecal occult blood tests were negative. A Meckel's scan, abdominal ultrasound scan, OGD and colonoscopy were normal. She underwent laparoscopy, which revealed a 2 cm hemangioma in the distal jejunum. A mini laparotomy was performed via the umbilicus to allow resection of the affected bowel segment and primary end-to-end anastomosis. She made an uneventful recovery and was found to have normal hemoglobin and hematinics at follow up. Histology confirmed a cavernous hemangioma extending through the full thickness of the bowel wall.

## DISCUSSION

Hemangiomas of the small bowel are rare tumors that often present with gastrointestinal bleeding. They are commonly found in the small bowel and hence often make diagnosis difficult. Initial investigations in the patient with gastrointestinal bleeding often include upper and lower gastrointestinal endoscopy, barium contrast study, and a Meckel's scan. These investigations are often normal and while useful in excluding other diagnoses, do not provide clear indication of a hemangioma. Other investigations such as red blood cell scan and angiography have a rather low yield when the vascular malformation is not actively bleeding and one may have to resort to exploratory laparotomy to determine the presence and location these tumors.[1,3]

Laparoscopy as employed in our two cases allows a diagnosis to be made without performing a laparotomy. The laparoscopy identifies the affected segment of bowel and if open excision is subsequently required, allows delivery to a minimally extended umbilical port site. Resection and anastomosis is then performed in the usual manner. A port site is positioned at the umbilicus for the laparoscope, with two lower abdominal ports for instruments. The small bowel is examined from the ileocecal junction proximally. Laparoscopy can be performed in conjunction with upper and lower gastrointestinal endoscopy under the same anesthetic, as in our first case. Prior insufflation of the gastrointestinal tract during endoscopy did not create any difficulties for the laparoscopy or identification of the pathology in this case. The avoidance of an open laparotomy helps to reduce post-operative analgesic requirement and achieves an early return of bowel function. Furthermore, there is the added cosmetic benefit of the small umbilical scar in girls.

Laparoscopy in these two cases also allowed a thorough examination of the whole length of small bowel using the ‘walking’ maneuver. The good light source and magnification enabled easy viewing of any mesenteric or seromuscular abnormalities. We did not proceed to intra-operative small bowel endoscopy as we felt that additional large hemangiomas were unlikely. We were also confident that the resected specimen had had a recent bleed because of the eroded appearance of the mucosal surface of the hemangioma when examined intra-operatively. We believe laparoscopy compliments other modalities in the management of occult gastrointestinal bleeding and the use of various modalities or in combination can be considered depending on the circumstances.
